# Conspecific and Heterospecific Information Use in Bumblebees

**DOI:** 10.1371/journal.pone.0031444

**Published:** 2012-02-08

**Authors:** Erika H. Dawson, Lars Chittka

**Affiliations:** Biological and Experimental Psychology Group, School of Biological and Chemical Sciences, Queen Mary University of London, London, United Kingdom; Università di Parma, Italy

## Abstract

Heterospecific social learning has been understudied in comparison to interactions between members of the same species. However, the learning mechanisms behind such information use can allow animals to be flexible in the cues that are used. This raises the question of whether conspecific cues are inherently more influential than cues provided by heterospecifics, or whether animals can simply use any cue that predicts fitness enhancing conditions, including those provided by heterospecifics. To determine how freely social information travels across species boundaries, we trained bumblebees (*Bombus terrestris*) to learn to use cues provided by conspecifics and heterospecific honey bees (*Apis mellifera*) to locate valuable floral resources. We found that heterospecific demonstrators did not differ from conspecifics in the extent to which they guided observers' choices, whereas various types of inorganic visual cues were consistently less effective than conspecifics. This was also true in a transfer test where bees were confronted with a novel flower type. However, in the transfer test, conspecifics were slightly more effective than heterospecific demonstrators. We then repeated the experiment with entirely naïve bees that had never foraged alongside conspecifics before. In this case, heterospecific demonstrators were equally efficient as conspecifics both in the initial learning task and the transfer test. Our findings demonstrate that social learning is not a unique process limited to conspecifics and that through associative learning, interspecifically sourced information can be just as valuable as that provided by conspecific individuals. Furthermore the results of this study highlight potential implications for understanding competition within natural pollinator communities.

## Introduction

The use of information provided by conspecific individuals is a widespread phenomenon found across a wide range of species. Traditionally social learning has concentrated almost exclusively on learning between members of the same species and interactions between heterospecific species have largely been overlooked. However, recent research has suggested that if different species overlap in their habitats, resources or predators, then the cues provided by heterospecific species could serve to be just as useful as those made available by conspecifics [1], [2]. Moreover heterospecific information has been recognised as having important ecological implications for community structure and formation, highlighting the importance of fully understanding this aspect of social information use [3].

Several pollinating insect species have been shown to use the cues provided by conspecifics in order to maximise foraging effort [4], [5], [6], [7]. However, less evidence exists for the utilisation of information provided by heterospecific species. As many pollinating insects share the same resources [8], [9], heterospecific information may be just as valuable as that provided by conspecifics. Many social learning phenomena can be explained by relatively simple cognitive processes, such as associative learning [2], [10], [11], whereby an animal learns that the presence of a conspecific predicts, for example, a reward or a predation threat. There should, however, be no reason why the unconditioned stimulus in such a learning process has to be provided by a conspecific exclusively. As long as the stimulus is reinforced by a reward or punishment, the conditioned stimulus could just as well be provided by a different species. Bees are able to learn even remarkable ecologically irrelevant stimuli (e.g. human faces [12]) as predictors of reward and therefore it would seem likely that they could also learn to use the cues provided by heterospecific pollinators. However, what is less clear is whether cues provided by different species are as equally salient as those from conspecifics. Leadbeater & Chittka [11] suggest that conspecific information may have a stronger inherent influence than information provided by heterospecifics, which could later be modified by experience. This study aims to address this question by investigating whether invertebrates show a better ability for learning conspecific cues over heterospecific cues. We conducted laboratory experiments with bumblebees (*Bombus terrestris*) to determine whether, and to what extent, subjects respond differentially to the visual social cues provided by conspecifics, heterospecific honeybees (*Apis mellifera*) and various non-social cues in a foraging context. In addition to this, we assessed to what degree previous social experience influenced social learning efficiency.

## Materials and Methods

### Experiment 1: Bees that had Previously Foraged with Conspecifics

#### (a) Test Subjects & Arena

Bumblebee colonies were obtained from Syngenta Bioline Bees (Weert, the Netherlands). Six colonies were used throughout Experiment 1. Each colony was housed in a wooden nest box (28×16×11 cm) that was connected to a flight arena (105×72×30 cm) by a Plexiglas tube. Since bumblebees are sometimes reluctant to land on new flower types [13], foragers were allowed to familiarise themselves with, and feed from, the experimental stimulus, a single artificial yellow flower. The flower was positioned at the entrance of the flight arena filled with sucrose solution 50% (v/v). In keeping with previous social learning bee experiment protocols (e.g. [14], [15], [16], [17]), foragers were allowed to feed from this flower with other workers. Once a motivated forager was identified, it was assigned to one of the five treatment groups: Conspecific; Heterospecific; Non-social (coin); Non-social (plastic disc); or No Cue.

#### (b) Learning phase

The single flower was then replaced with eight yellow artificial flowers (35 mm diameter, craft foam circles, placed on top of glass vials, 50 mm in height) that were randomly placed around the arena. For bees in the Conspecific treatment group, a single dead (freshly freeze-killed), *B. terrestris* worker, taken from an unrelated colony, was placed in a foraging position on four of the eight flowers in the arena. These cue occupied flowers were rewarded with 25 µl of 50% (v/v) sucrose solution. The remaining four flowers were unoccupied and contained no reward. For the Heterospecific treatment, rewarding flowers were occupied by a single dead heterospecific species, a honeybee (*Apis mellifera*). Individuals used to provide social cues had been killed by placing them at −20°C a day before experimentation and defrosted at room temperature just before testing took place. Note that bees' visual spatial resolution is too poor to distinguish visually between a motionless worker sitting on a flower and a dead bee [18], and previous tests on within-species social learning have proven pinned, dead specimen to be readily acceptable by bumblebee workers choosing flowers [19].

To explore whether any arbitrary cue associated with floral rewards might perform the same function as a social cue, we also used a variety of other visual cues comparable in size to the social cues. For these Non-social treatments, two groups of bees were trained with a different visual cue each: a five pence coin, 8 mm diameter (Non-social (coin)) and a white styrene plastic disc, 8 mm diameter (Non-social (plastic disc)). In all trials with the No cue group, no cues were used and all eight flowers were rewarding. Bee subjects were allowed three foraging bouts during the learning phase, with the rewards in the cue associated flowers being replenished after each bout. The position of all eight flowers was changed after each bout to ensure that subjects did not simply learn the location of the rewarding flowers.

#### (c) Test 1: Yellow Flowers with & without Cues

Testing took place straight after the third bout of the learning phase. All flowers were replaced with eight ethanol cleaned yellow flowers to eliminate any scent cues that may have remained from previous visits. Again, with exception of the No cue treatment, four of these flowers had a cue attached while the remaining four had no attached cue. “Demonstrators” were also replaced with new dead specimens; non-social cues were cleaned with ethanol prior to tests. None of the flowers were rewarding to ensure that the number of visits reflected the subject's preference and was not just a result of revisiting rewarding flowers. To assess whether bee subjects had learned to associate the specific cue with a reward, the number of visits to cue occupied and unoccupied flowers was recorded. A visit was defined as the subject landing on the flower. The test ended once the subject left the arena to return to the hive.

#### (d) Test 2: Transfer Test with Blue Flowers

The second test ascertained whether bee subjects could then transfer the information that they had learnt in the learning phase to a new flower “species”. Immediately after test 1, cue occupied yellow flowers were rewarded again for a single foraging bout to reinforce the association that had occurred in the learning phase. Once the bee subjects returned to the hive to offload the sucrose solution, all yellow flowers were replaced with a new flower “species”; artificial blue flowers (35 mm diameter, craft foam circles, placed on top of glass vials, 50 mm in height). These new flowers were randomly distributed throughout the arena, with the appropriate cues attached. Again all the flowers were unrewarded. Since subjects only ever landed on cue occupied flowers, recording the proportion of landings on cue occupied flowers did not give an informative indication of how well bees identified their respective cues on the new flower colour. For this reason, the time for each subject to land on the first blue cue occupied flower was recorded. The test finished when the subject left the arena.

#### (e) Analyses

To establish whether subjects in each treatment group learnt to associate their specific cue with a reward, the proportion of visits to occupied flowers in test 1 was compared against the chance expectation of visits to cue occupied flowers (0.5) using a two-tailed binomial test. To assess learning performance between the different treatment groups in test 1, the proportion of visits to cue occupied flowers was compared between treatments using a generalised linear model with a quasi-binomial error distribution to correct for overdispersion. Only the first eight landings made by subjects were analysed. The No Cue treatment was excluded from this analysis as no cues were used and therefore proportion of landings to cue-occupied flowers could not be calculated.

A survival analysis using non-parametric Cox proportional hazard models was used to analyse latency times between treatment groups in test 2. Ten bees were tested within each treatment group. Subjects that made less than eight landings in test 1 were excluded from both analyses (Conspecific n = 10; Heterospecific n = 10; Non-social (coin) n = 10; Non-social (plastic disc) n = 10; No cue n = 10). All statistical analyses were carried out using the R statistical software (v.2.12.0).

### Experiment 2: Bees that had no Prior Social Foraging Experience

To ensure that the social pre-training conditions that test bees experienced in Experiment 1, whereby foragers were allowed to feed with nest mates prior to experimentation, did not predispose bees to learn conspecific cues significantly better than heterospecific and non-social cues, the experiment was repeated, however, this time ensuring that test bees had absolutely no previous foraging experience with conspecifics. To do this, only foragers newly emerged from the pupae were selected for experiments to control for any previous social foraging experience. The hive was fed by administering 50% (v/v) sucrose to honeypots and the colony was kept in complete darkness so as to avoid visual associations with rewarding sucrose and conspecifics.

We also decided to use a more prominent, 3-dimensional non-social cue to ensure that any non-social cue effect in Experiment 1 was not a direct result of less salient properties of the non-social cues. In Experiment 2 we used a 3-dimensional wooden rectangular cuboid (14×6×6 mm) painted with a black paint that had the some low reflectance across the bee visual spectrum as the black body parts of a *B*. *terrestris* forager [20]. Asides from these elements, all procedures were kept the same as in Experiment 1. Fifteen bees were tested within each treatment group, but subjects that made fewer than eight landings in test 1 were excluded from both analyses (Conspecific n = 13; Heterospecific n = 13; Non-social (wooden cuboid) = 12; No cue n = 15).

## Results

### Experiment 1: Bees that had Previously Foraged with Conspecifics

#### (a) Test 1: Yellow Flowers with and without Cues

**Figure 1:**
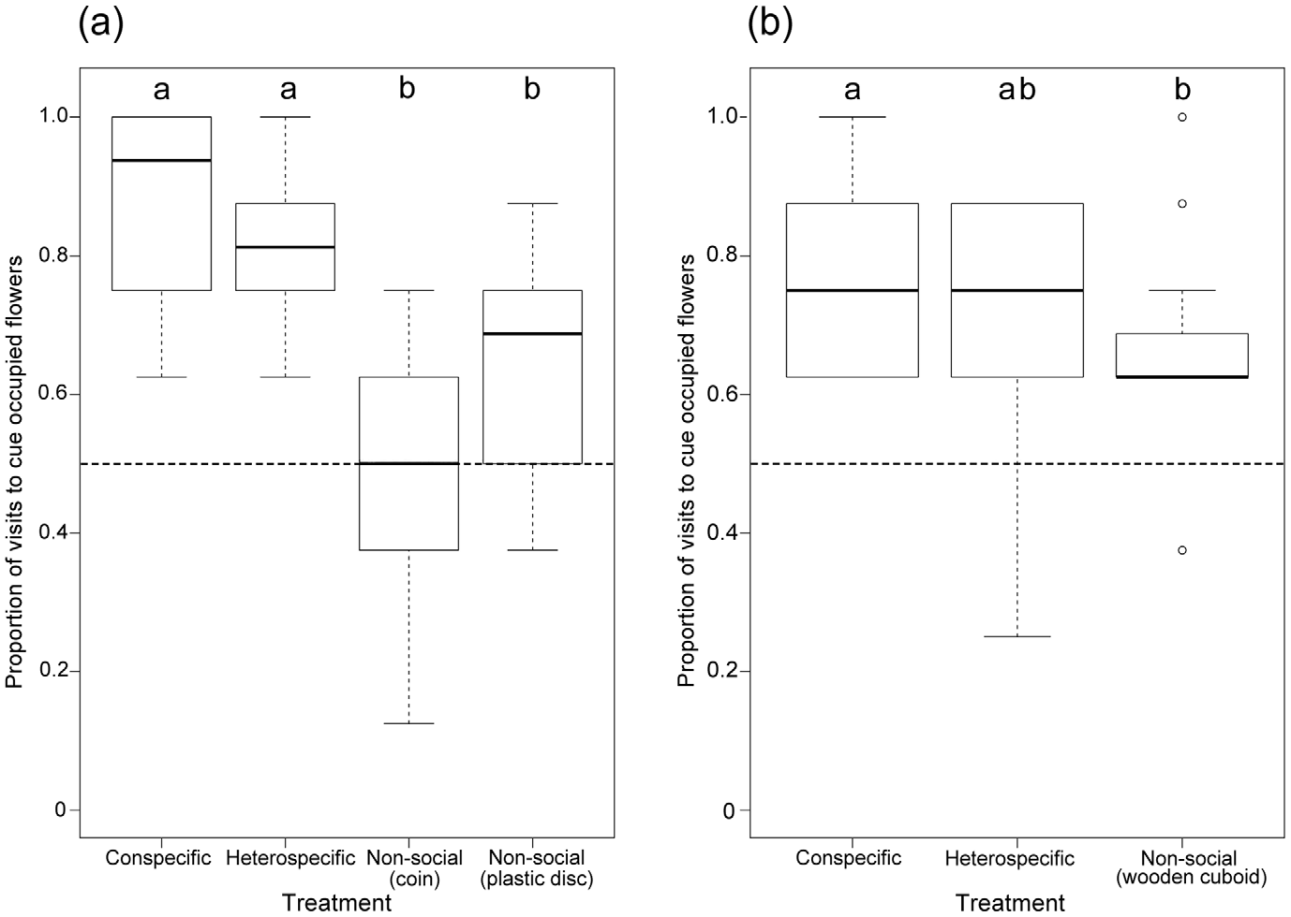
Proportion of visits to cue occupied yellow flowers. (a) Proportions shown for bees that were allowed to forage with conspecifics prior to experimentation and (b) proportions shown for bees that had never had any social foraging experience. Medians, interquartile range and maximum/minimum values are indicated. The dashed line (0.5) signifies the chance expectation of landing on cue occupied flowers (i.e. no learning has occurred). There was no difference in proportions between the Conspecific and Heterospecific groups, but all Non-social groups performed significantly worse than the Conspecific group.

All treatment groups, with the exception of the Non-social (coin) group, learned to associate a reward with their respective cues (Fig. 1(a), two-tailed binomial test *p* = 1; *p* = <0.001; *p* = <0.001; *p* = <0.001 for treatments Non-social (coin), Conspecific, Heterospecific and Non-social (plastic disc) respectively). There was no significant difference in the proportion of landings on occupied flowers between the treatments that had bumblebees or honeybees as demonstrators (Fig. 1(a); Conspecific vs. Heterospecific: *E* = −0.4796, T-value = −0.987, *p* = 0.33) and between the Non-social treatment groups (Non-social (coin) vs. Non-social (plastic disc): *E* = 0.619, T-value = 1.743, *p* = 0.09). However there was a significant difference in learning performance between the social cues and the non-social cues (Fig. 1(a) Conspecific vs. Non-social (coin): *E* = −1.9459, T-value = −4.378, *p* = <0.001; Conspecific vs. Non-social (plastic disc): *E* = −1.3269, T-value = −2.941, *p* = <0.01; Heterospecific vs. Non-social (coin): *E* = −1.4663, T-value = −3.680, *p* = <0.001; Heterospecific vs. Non-social (plastic disc): *E* = −0.8473, T-value = −2.088, *p* = <0.01).

#### (b) Test 2: Transfer Test with Blue Flowers

**Figure 2:**
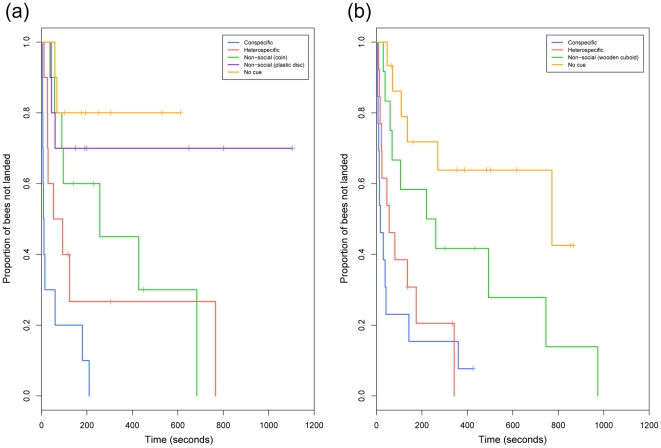
Transfer Test: Kaplan-Meier curves of latency times to land on blue flowers. (a) Curves shown for bees that were allowed to forage with conspecifics prior to experimentation and (b) curves shown for bees that had never had any prior social foraging experience. Each step represents the time at which a bee landed and crosses throughout curves indicate where censoring occurred i.e. where a test subject left the arena without making any landings. For example, graph (a) shows that 80% of subjects within the Heterospecific group (red line) landed on a flower and all bees that did land, landed within 766 seconds. In (a), the Conspecific group had significantly shorter latency times than the Heterospecific group, whereas Conspecific and Heterospecific groups had similar latency times in (b). The Conspecific group had shorter latency times than the Non-social groups.

Test 2 assessed how readily subjects would accept a novel (blue) flower type depending on which previously learnt cues were presented on the flowers, by assessing latency time to land on cue occupied blue flowers. There was a clear significant difference in latency times between the social treatments whereby test subjects within the Conspecific treatment performed significantly better than subjects within the Heterospecific group (Fig. 2(a), Z-value = −2.16, *p* = <0.05). The Conspecific treatment group also significantly outperformed subjects within all other treatment groups (Conspecific vs. Non-social (coin): Z-value = −2.963, *p* = <0.01; Conspecific vs. Non-social (plastic disc): Z-value = −3.565, *p* = <0.001; Conspecific vs. No cue: Z-value = −3.490 *p* = <0.001). The Heterospecific treatment group performed significantly better than the Non-social (plastic disc) and No cue treatment groups (Z-value = −2.017, *p* = <0.05; Z-value = −2.075, *p* = <0.05 respectively) but had similar latency times to the Non-social (coin) group (Z-value = −0.840, *p* = 0.4). The two Non-social treatment groups took similar times to land (Z-value = −1.321, *p* = 0.19) and latency times for both Non-social treatment groups did not differ significantly from the No cue group (Non-social (coin) vs. No cue: Z-value = −1.512, *p* = 0.13; Non-social (plastic disc) vs. No cue: Z-value = −0.312, *p* = 0.755).

### Experiment 2: Bees that had no Prior Social Foraging Experience

#### (a) Test 1: Yellow Flowers with and without Cues

Subjects in the social Conspecific, Heterospecific and Non-social (wooden cuboid) treatment groups both learnt to associate a reward with their respective cues (Fig. 1(b); two-tailed binomial test *p*<0.001; *p*<0.001; *p*<0.01 for treatments Conspecific, Heterospecific and Non-social (wooden cuboid) respectively). As in Experiment 1, we found no significant difference in learning performance between subjects trained with conspecific and heterospecific cues (Fig. 1(b); *E* = −0.6522, T-value = −1.789, *p* = 0.0823) indicating that both conspecific and heterospecific cues were learnt equally well. Again subjects within the Conspecific treatment made more landings on cue occupied flowers than subjects within the Non-social (cuboid) treatment group (Fig. 1(b); *E* = −0.7735, T-value = −2.103, *p* = <0.05). However, the Heterospecific treatment group did not differ significantly from the Non-social (wooden cuboid) group (Fig. 1(b); *E* = −0.1214, T-value = −0.358, *p* = 0.722).

#### (b) Test 2: Transfer Test with Blue Flowers

When faced with a novel blue flower type, subjects in the Conspecific treatment group had very similar latency times to subjects in the Heterospecific treatment group (Fig. 2(b); Z-value = −1.023, *p* = 0.307), but significantly shorter latency times than subjects in the Non-social (wooden cuboid) and No cue treatment groups (Fig. 2(b); Z-value = −2.685, *p* = <0.01; Z-value = −3.923, *p* = <0.001 for treatments Non-social (wooden cuboid) and No cue respectively). This makes it likely that the slight preference for conspecific cues observed in the transfer test in Experiment 1 was merely a result of the subjects' exposure to conspecifics before experiments began. Visual inspection of Figure 2(b) shows that whether heterospecific or conspecific demonstrators were present, subjects landed with very similar latencies, whereas the non-social (wooden cuboid) cue was less readily accepted. However, the Heterospecific treatment latency times did not quite differ statistically from the Non-social (wooden cuboid) treatment at the 5% level (Fig. 2(b); Z-value = −1.712, *p* = 0.087) but were significantly shorter than the No cue treatment (Z-value = −3.05, *p* = <0.01). The Non-social (wooden cuboid) group had similar latency times to the No cue group (Z-value = −1.568, *p* = 0.12), and thus was clearly a less efficient cue despite it having signalled reward with the same reliability as the social cues during the previous training on yellow flowers.

## Discussion

We assessed whether bumblebees learn heterospecific appearance on flowers as predictors of reward to the same degree as information provided by members of their own species, and found that this was indeed the case. This raises the question of whether any arbitrary cue that is reliably associated with the same outcome might be used with equal probability. However, we found that as opposed to cues provided by heterospecific demonstrators, non-social cues bearing the same information were consistently less efficient than conspecifics as pointers to rewarding flowers. This was true irrespective of the colour, shape, and texture of the non-social cues that we tested. Bumblebee workers appear to have a preparedness for accepting cues with a pollinator-like appearance over other cues that might in nature simply be part of the flower display.

The same overall picture holds in a transfer test, where subjects were faced with a novel target flower colour that they had never seen before. The only familiar cues on these new flowers were those that subjects had previously been exposed to in association with rewarding yellow flowers. In the transfer test, subjects most swiftly accepted flowers occupied with conspecific demonstrators, closely followed by flowers with heterospecific demonstrators on them, which in turn as an overall trend, were more efficient than non-social cues; when the novel flowers bore no familiar cue, subjects hardly visited them at all over the testing period. In this transfer test, the difference in the efficiency of conspecific and heterospecific demonstrators vanished, however, when we tested subjects that had recently emerged from the pupae and which were entirely prevented from foraging alongside conspecifics prior to the experiment.

The difference in learning efficiency as a result of pre-training protocol highlights an important issue in social learning experiments. In studies from flower-choice copying in bees to imitation in primates [21], wherever differences in the efficiency of different demonstrator species are observed, a crucial issue might be whether subjects were raised alongside conspecifics or members of different species, or whether they had no relevant exposure to either before the beginning of test.

**Figure 3:**
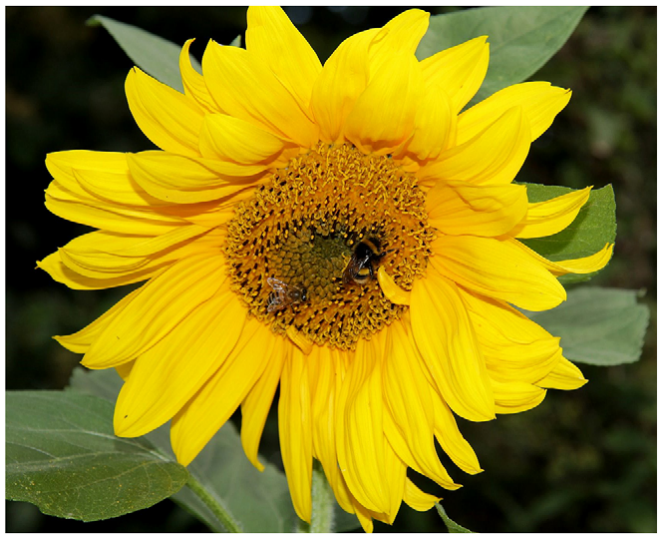
A bumblebee (*Bombus terrestris*) and a honeybee (*Apis mellifera*) foraging from the same sunflower inflorescence (*Helianthus annuus*). Photograph by A. Jaithirtha.

Whether bumblebees use and learn heterospecific sourced information to the same degree as conspecific information in the wild requires further investigation. However, the conditions in our experiment without pre-training to social cues most likely represent those of a wild colony, where foragers emerge singly from the colony and fly long distances to flowers [22]. In such conditions, foragers are unlikely to encounter conspecific foragers on flower patches exclusively. Therefore it seems likely that wild bees would have opportunities to learn heterospecific and conspecific information to an equal degree. Since generalist pollinators such as those under investigation here typically share many flower species [8], [9] , using information provided by heterospecific species could often help insects identify rewarding flowers. This is especially the case for inflorescences that contain many nectaries in a single display, such as sunflowers (*Helianthus annuus*), that will often be fed from by multiple pollinators simultaneously [19] (Fig. 3.) As opposed to some stingless bees [17], bumblebees are not known to engage in active interference competition; they do not displace each other from flowers by overt aggression.

In addition to this, we also demonstrated that heterospecific cues, once they have been learnt as predictors of reward on one flower species, can facilitate the sampling of new flower species.

Since our findings clearly demonstrate that information travels freely across species boundaries, this may have important implications for understanding competition in natural pollinator communities. Competition may be much more severe than previously assumed if individuals not only use individual exploration and copying of conspecifics to identify rewarding plants [7], but also use the information provided by a competing species. Our findings imply that information spreads swiftly across pollination systems and the subsequent necessity to explore alternative food resources could be much faster than expected. This could well have profound implications for pollination services and the competition between sympatric plant species.

In conclusion, we have demonstrated that under similar learning conditions, heterospecific social learning is not only possible but also as efficient as that of conspecific social learning. Moreover, this is the first experimental study to demonstrate that feeding heterospecifics can be used by bumblebees to locate familiar and new food sources and highlights the implications for competition within natural pollinator communities.
